# Intermittent theta burst stimulation enhances prefrontal activation and connectivity: evidence from fNIRS

**DOI:** 10.3389/fnhum.2025.1608502

**Published:** 2025-08-26

**Authors:** Yi-ning Zhao, Xing-yu Zhang, Ying-ying Huang, Ji-chun Wu, Xia Bi

**Affiliations:** ^1^Department of Rehabilitation Medicine, Shanghai University of Medicine and Health Sciences Affiliated Zhoupu Hospital, Shanghai, China; ^2^Department of Sport Rehabilitation, Shanghai University of Sport, Shanghai, China; ^3^Graduate School of Shanghai University of Traditional Chinese Medicine, Shanghai, China

**Keywords:** transcranial magnetic stimulation, near-infrared brain imaging, functional connectivity, dorsolateral prefrontal cortex, functional near-infrared spectroscopy (fNIRS)

## Abstract

**Objective:**

To investigate the neuro-regulatory mechanisms of intermittent theta burst stimulation (iTBS) on prefrontal brain function.

**Methods:**

Functional near-infrared spectroscopy (fNIRS) was used to monitor the blood flow dynamics response in the prefrontal cortex of 20 healthy adults. Measurements were taken at five time points (10, 25, 40, 55, and 70 min) after iTBS stimulation, as well as before stimulation (T0). The activation intensity of prefrontal cortex was assessed by quantifying the relative change in oxygenated hemoglobin concentration (β value), and functional connectivity between prefrontal-related brain regions was evaluated by calculating the correlation coefficients of oxygenated hemoglobin concentration between channels across time series for each subject.

**Results:**

Compared with the pre-stimulation period, the activation intensity in the prefrontal cortex was significantly higher from 10 to 70 min after iTBS stimulation. Specifically, seven channels showed statistically significant differences, with peak effects occurring 10 min after intervention and gradually attenuating over time. Additionally, compared with T0, the functional connectivity (FC) strength of the prefrontal network was markedly enhanced 10 min after intervention, accompanied by a notable increase in the number of connections between channels. However, the FC strength gradually weakened over time, and no statistically significant differences in FC strength were observed at 55 and 70 min post-intervention. Taken together, We conclude that the neural modulation effects of a single iTBS session persist for ~40 min. These results elucidate the regulatory effects of iTBS intervention on brain functional activity from the perspective of brain functional connectivity, providing reference and evidence for the clinical application of iTBS.

## 1 Introduction

Since 1985, when magnetic stimulation technology was introduced, repetitive transcranial magnetic stimulation (rTMS) has become a key tool for treating neuropsychiatric disorders. Intermittent theta-burst stimulation (iTBS), a new rTMS approach, offers significant advantages in treating depression (Kong et al., 2023), drug addiction (Su et al., 2020), and neurodegenerative disorders (Wu et al., 2024). It shortens treatment time to just 3 min, compared to 20–30 min for conventional rTMS (Lefaucheur et al., 2020), while maintaining or even enhancing neuromodulatory effects (Blumberger et al., 2018). This advantage likely arises because iTBS simulates the neuroplasticity mechanism associated with hippocampal long-term potentiation (LTP), which leads to lasting changes in synaptic strength through a rhythmic pattern of 50-Hz triple-pulse clusters separated by 5-Hz intervals (Hu et al., 2025).

The left dorsolateral prefrontal (DLPFC) region plays an important role in cognition and emotion regulation and is widely considered a classical stimulation area for improving mood and cognitive function (Webler et al., 2022), and abnormal functional connectivity is closely associated with cognitive deficits in a wide range of neuropsychiatric disorders (Dong et al., 2023). In the past decade, (Guse et al. 2010) reviewed studies on rTMS aimed at improving cognition in patients. They found that high-frequency rTMS (5, 10, or 15 Hz) applied with an intensity of 80%−110% of the motor threshold over 10–15 consecutive sessions targeting the left DLPFC for 1–2 weeks was more effective in enhancing cognitive function. Additionally, 20 Hz rTMS applied to the left DLPFC significantly improved cognitive function in patients with Alzheimer's disease (AD) after 6 weeks (Li et al., 2021). However, the neural mechanisms through which rTMS enhances clinical symptoms and cognitive function in neurological disorders remain intricate and not fully understood.

However, most of the existing studies focus on the behavioral effects of iTBS and lack in-depth exploration of its neurovascular coupling mechanism and brain network dynamics. In contrast, functional near-infrared spectroscopy (fNIRS) is a non-invasive optical imaging technique that offers several advantages: low cost, portability, resistance to motion interference, good compatibility, and high temporal and spatial resolution (Pinti et al., 2020). This makes it uniquely suited for real-time monitoring of the DLPFC's hemodynamic response (Jiang et al., 2023). (Struckmann et al. 2022) utilized fNIRS and functional magnetic resonance imaging (fMRI) to evaluate the functional connectivity network following iTBS treatment for depression. They found that patients experienced significant improvement in symptoms after the iTBS intervention, which was linked to reduced connectivity between the left insula and the left dlPFC. Research indicates that (Liu et al., 2023) fNIRS can assist in screening and diagnosing Post-ischemic Stroke Executive Impairment (PISEI). After a single session of high-frequency transcranial magnetic stimulation to the left DLPFC, patients with PISEI showed significant improvements in both the time taken and the number of errors made on the Stroop test. Functional activity in brain regions was significantly active, and the strength of functional connectivity was significantly increased.

This study innovatively combines single-session iTBS intervention with multi-channel fNIRS monitoring. We applied iTBS stimulation to the left DLPFC region of healthy young adults and collected fNIRS data before and after the intervention. Subsequently, we analyzed changes in cognitive-related brain network functional connectivity patterns and brain network activation before and after iTBS intervention. The study aims to elucidate the regulatory effects of iTBS on brain functional activity from the perspective of brain functional connectivity, providing reference and evidence for its clinical application.

## 2 Methods

### 2.1 Participants

A total of 20 college students from the Shanghai Medical College of Health Sciences were selected for this study. The participants were 10 males (22.80 ± 0.81years) and 10 females (23.13 ± 0.92 years). The inclusion criteria are as follows: (1) Absence of any previous neurological or psychiatric diseases or other health problems; normal intelligence; (2) Age between 18 and 26 years (Sowell et al., 2003; Tamnes et al., 2010); (3) Right-handedness (Knecht et al., 2000; Beam et al., 2009); (4) Absence of contraindications to rTMS; (5) Informed consent was signed before the experiment. The exclusion criteria are as follows: (1) Administration of psychotropic drugs or neuroactive substances within the last 1 month; (2) intake of alcohol, caffeine >200 mg/day, or nicotine products 48 hours prior to enrolment; (3) presence of contraindications to rTMS (e.g., pacemakers, cochlear implants, and other metallic implants *in vivo*); (4) Female subjects are in pregnancy or on days 7–14 of the menstrual cycle (fluctuations of oestrogens during the luteal phase may affect fNIRS signals) (Xia et al., 2007; Chen D. Y. et al., 2023).

### 2.2 fNIRS data acquisition

The prefrontal brain regions of the subjects were monitored using a 37-channel near-infrared functional brain imaging system, the BS-7000 (Wuhan Yiruide, China). The headcap probe is based on the 10–20 international standard lead design and consists of 12 transmitting optodes and 12 receiving optodes, which collectively constitute 37 acquisition channels. These channels are used to capture data from the subject's brain network. The distance between the probe and the light source is 30mm. The brain area below the midpoint of the probe and the light source is the primary probing area of the channel, which is calibrated concerning the Automated Anatomical Labeling (AAL). A template for labeling cerebral cortical subdivisions of the channel was employed, along with recording near-infrared light intensity signals at different wavelengths (690, 830 nm) at a sampling frequency of 100 Hz.

**Figure 1:**
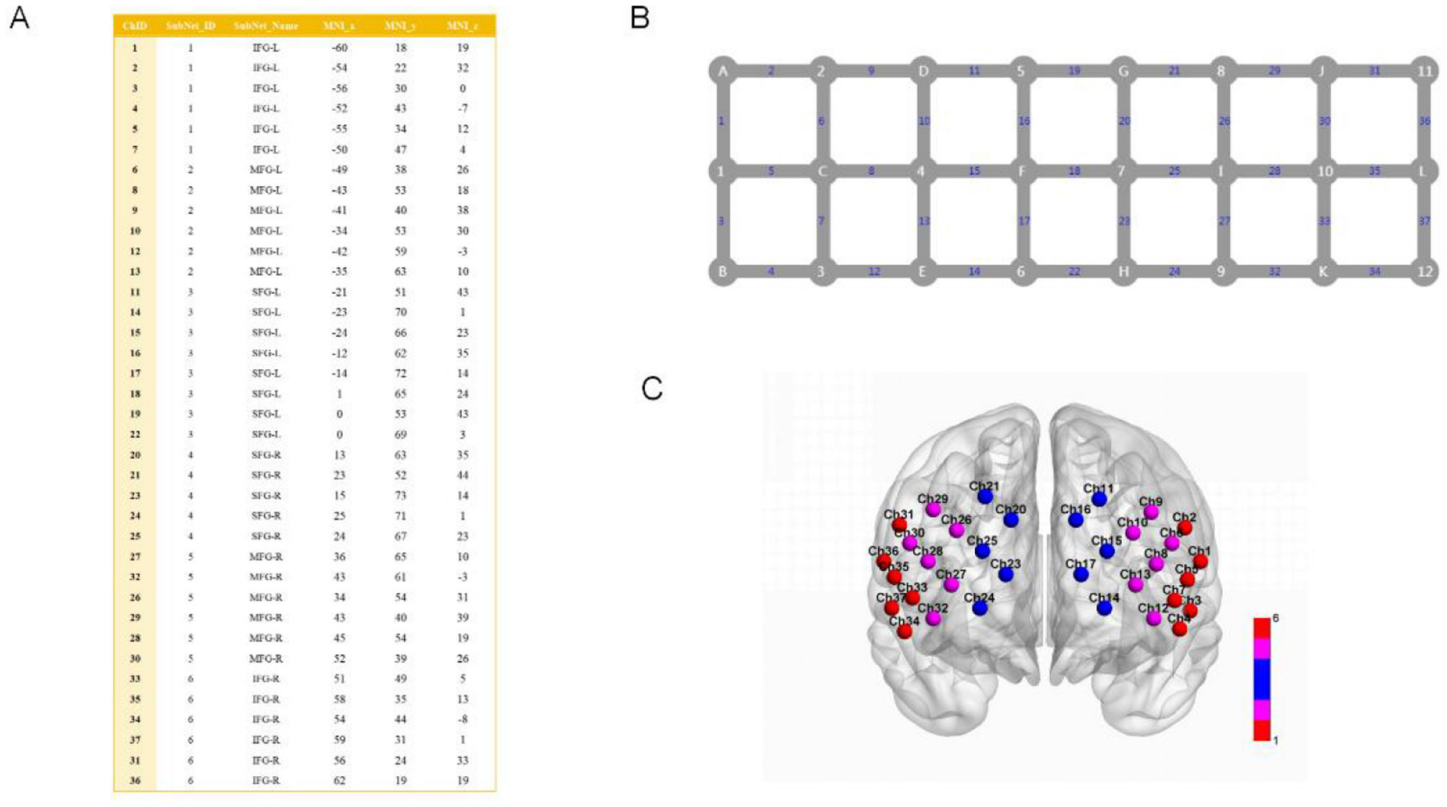
Channel parameters, scalp layout, and brain region mapping used in this experiment. **(A)** Parameter table for the 37 channels corresponding to the AAL brain region atlas in the fNIRS experiment, where ChID denotes the channel number. SubNet ID is the subnetwork ID, which divides the 37 channels into 1–6 different subnetworks. SubNet Name is the subnetwork name, based on anatomical or functional brain region naming, corresponding to the AAL brain region classification system. MNI *x*, MNI *y*, MNI *z*: three-dimensional coordinate values based on the Montreal Neurological Institute (MNI) standard brain coordinate system, reflecting the position of the channel in standard brain space. The method for mapping channels to regions is referenced from previous studies (Chen H. et al., 2023). **(B)** Schematic diagram of the 37-channel layout and connection relationships. Gray nodes labeled A–L represent the emitters of the fNIRS device, while numbers 1–12 represent the detectors of the fNIRS device. Blue numbers 1–37 represent detection channels. **(C)** Visualization of the 37-channel distribution of the prefrontal brain region in fNIRS. Colored spheres (Ch + number) represent fNIRS detection channels, including bilateral SFG (red), bilateral MFG (pink), and bilateral LFG (blue).

In this study, the primary observation area was the prefrontal lobe, which was covered by the fiber optic cap channel and divided into six brain regions according to the AAL brain regions. These were the bilateral superior frontal gyrus (SFG), middle frontal gyrus (MFG), and lower frontal gyrus (LFG). The results of the localization are shown in Figure 1.

The subject remained relaxed and wore a head cap after sitting quietly for 5 min in a comfortable position. During the scan, the subject remained relaxed with their eyes closed but did not fall asleep, and resting state data were collected.

### 2.3 Design of experiments

**Figure 2:**
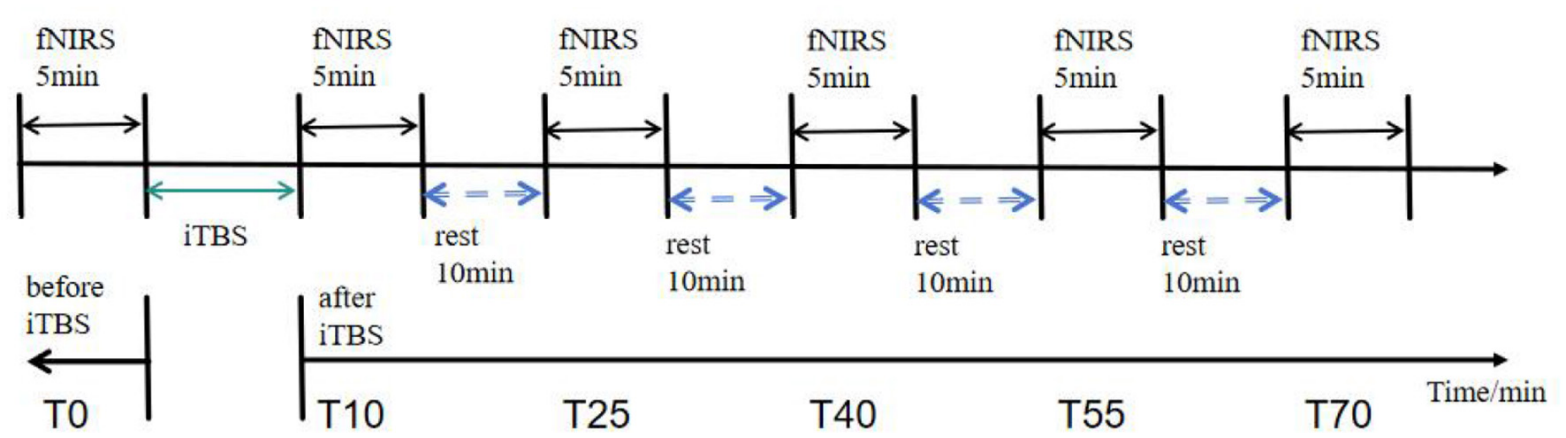
fNIRS data acquisition workflow diagram. Resting-state data were collected for 5 min at 5 min before the iTBS intervention (T0) and at 10, 25, 40, 55, and 70 min after the intervention (T10, T25, T40, T55, and T70).

This study employed a single-factor, six-level within-subjects design with two independent variables: intervention type—intermittent theta burst stimulation (iTBS)—and time point, which includes a baseline period (pre-TMS: T0) and five post-intervention time windows (post-TMS: 10, 25, 40, 55, and 70 min). Each complete module consists of a 30-s preparation period, a 5-min resting-state recording, and a 30-s recovery period. The blocking interval between modules is 10 min (Figure 2).

### 2.4 TMS intervention

A YRD-CCY-I transcranial magnetic stimulation device (Iridium, Wuhan, China), with a 70-mm figure-of-eight coil and a maximum output magnetic field of 3.0 T, was employed to stimulate the DLPFC using a magnetic stimulation cap to guide coil placement. Before stimulation, the subject was comfortably positioned either supine or seated, with the entire body relaxed.

Before stimulation, the motor threshold (MT) was measured for each subject. The coil was placed in the left M1 area, tangential to the scalp, and the myoelectric amplifier was placed in the right thenar muscle to record the motor evoked potentials, with the anode affixed proximally, the cathode distally, and the ground wire affixed to the radial styloid process. Subjects exhibiting a relaxed state of the target muscles were administered a minimum of 10 consecutive single-pulse stimuli using TMS to ascertain the resting motor threshold (rMT). The rMT was defined as the minimum stimulus intensity that excites a wave amplitude >50 μV MEP at least five out of 10 stimuli, expressed as a percentage of the maximum output intensity of the stimulator.

Place the figure-eight coil tangentially on the skull surface, position its center over the left DLPFC, and set the stimulation intensity to 80% RMT. iTBS stimulation parameters: intra-cluster frequency 50 Hz, number of intra-clusters 3; inter-cluster frequency 5 Hz, number of inter-clusters 10; stimulation time 2s, interval time 8s; number of pulses 600; treatment time 200s.

### 2.5 fNIRS data processing

The raw data of 37 channels were preprocessed using Matlab software running the Homer2 toolbox: (1) the raw light intensity was converted to optical density data; (2) motion artifacts were identified to reduce the disturbing factors generated by body micro-movements; (3) motion artifacts were removed by bar interpolation to patch the data; (4) the interfering signals from external and self-factors were removed by band-pass filtering from 0.01 to 0.10 Hz; (5) according to the modified Lambert–Beerlaw, the optical density data were converted into oxygenated hemoglobin (Oxy-Hb) concentration change values with a high signal-to-noise ratio; (6) the hemodynamic data of each block were superimposed and averaged. At the same time, we implemented strict signal separation techniques during preprocessing of fNIRS resting-state data. First, we used short-distance channels sensitive to superficial tissues to extract superficial hemodynamic signals. Then, we regressed these signals out of the target signals as noise sources to reduce contamination from extracranial components. Additionally, leveraging the characteristic that short light source-detector distance channels primarily reflect superficial tissue signals, we applied algorithms to correct long-distance channels, thereby isolating purer intracerebral hemodynamic information. These signal separation and correction techniques effectively enhance the signal-to-noise ratio of resting-state fNIRS data, providing a more reliable foundation for subsequent analysis. Subsequently, the data were subjected to analysis using the NIRS-KIT toolbox. Given that the signal-to-noise ratio of HbO_2_ is superior to that of HbR, only the obtained HbO_2_ data were employed in the subsequent data analysis (Cui et al., 2010; Wang and Lu, 2022; Luo et al., 2024).

Brain activation analysis: We performed task-like activation analysis on resting-state data to obtain brain region activation maps for each time point, based on previous literature (Chen D. Y. et al., 2023). A general linear model (GLM) was established for HbO_2_ change values, and the resulting activation coefficient β values represent changes in cortical neuron activity within the corresponding channel. The larger the β value, the higher the activation level.

Brain network functional connectivity strength analysis: all resting state FC analyses were performed using NIRS-KIT. Pearson correlation coefficients were calculated between all channel time series to determine FC between each pair of measurement channels. This process yielded a 37 × 37 connectivity matrix for each patient. BrainNet Viewer software was used to plot a 3D map of the brain functional network based on the Pearson correlation coefficient matrix. Additionally, a circle plot was generated using a Matlab script based on the same matrix.

### 2.6 Statistical analysis

This experiment utilized GraphPad 9.0 for data processing and analysis. The Shapiro-Wilk test was employed to assess data normality. Quantitative data with normal distribution are expressed as mean ± standard deviation (*x* ± *s*). Paired-sample *t*-tests or one-way analysis of variance (ANOVA) are conducted accordingly. A *p*-value < 0.05 was considered statistically significant. Brain network visualization images were created using the Brain Connectivity Toolbox in MATLAB. Multiple comparisons of β values, functional connectivity (FC), and Pearson correlation coefficients were corrected using the false discovery rate (FDR) method for all statistical tests.

## 3 Results

### 3.1 Differences in the activation of the prefrontal cortex before and after iTBS

**Figure 3:**
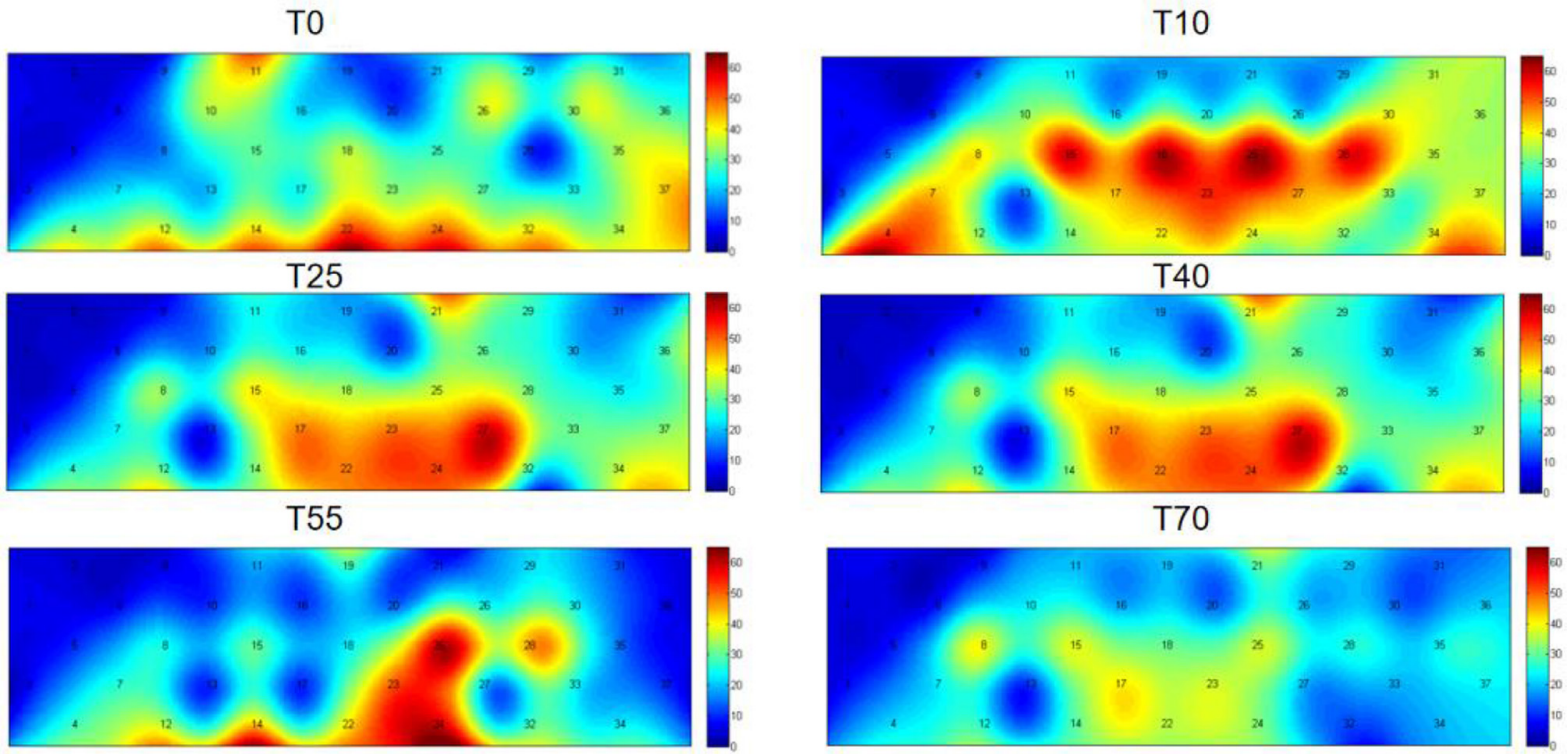
Two-dimensional plots of group-average HbO_2_ concentration changes in the prefrontal cortex before (T0) and 10, 25, 40, 55, and 70 min after iTBS stimulation. The cyan background color represents the baseline, with numbers 1–37 representing channels. While the warm colors (i.e., red) at the channel location indicate activated areas. The deeper the red color in an area, the greater the level of activation.

Functional near-infrared spectroscopy (fNIRS) was used to obtain blood oxygen activation heatmaps of the prefrontal cortex. In these heatmaps, blue-cool tones indicate low activation, while red-warm tones indicate high activation. Overall, the participants' prefrontal cortex showed different levels of activation at various time points. These times were both before and after iTBS stimulation. Compared to pre-stimulation (T0), the red-activated regions were more concentrated and larger in area at 10 min post-stimulation (T10), suggesting that iTBS temporarily enhanced task-related activation in the prefrontal cortex. Over time, the effects of iTBS gradually diminished: at T25 and T40, the red activation areas remained large, while at T55 and T70, these areas significantly decreased or even disappeared (Figure 3). A one-way analysis of variance was conducted to compare HbO_2_ β values across all channels and time periods (Table 1). The results showed significant differences in channels 4, 15, 18, 22, 24, 25, and 28.

**Table 1:** Comparison of Beta values in 37 channels of the prefrontal cortex at different time points before and after iTBS (Mean ± SD, × 10^−3^mol/mm).

**Channel**	**T0**	**T10**	**T25**	**T40**	**T55**	**T70**	***F*-value**	*p*-Value^*^	**Channel**	**T0**	**T10**	**T25**	**T40**	**T55**	**T70**	***F*-value**	*p*-Value^*^
CH1	7.37 ± 4.86	7.21 ± 4.10	5.86 ± 4.82	4.43 ± 5.85	6.04 ± 5.17	6.13 ± 4.24	0.929	0.465	CH20	7.14 ± 3.17	6.79 ± 1.44	6.96 ± 2.43	6.95 ± 2.86	6.26 ± 2.16	6.67 ± 2.49	0.309	0.907
CH2	5.68 ± 5.26	7.71 ± 7.59	7.88 ± 5.86	6.62 ± 8.18	8.90 ± 5.82	7.34 ± 6.78	0.553	0.740	CH21	6.59 ± 1.38	6.49 ± 1.49	6.14 ± 0.76	6.12 ± 0.54	6.04 ± 0.74	7.46 ± 5.96	0.824	0.535
CH3	7.18 ± 6.68	5.31 ± 5.83	6.71 ± 5.31	6.04 ± 5.50	7.74 ± 5.73	3.72 ± 5.77	1.243	0.294	CH22	6.94 ± 1.20	6.05 ± 0.53	6.14 ± 0.87	6.45 ± 0.60	6.42 ± 0.98	5.79 ± 0.86	4.519	0.012
**CH4**	6.24 ± 0.69	7.00 ± 1.10	5.93 ± 0.80	6.45 ± 0.91	6.27 ± 1.15	5.96 ± 0.87	3.548	0.005	CH23	6.30 ± 0.82	6.35 ± 0.52	6.15 ± 0.86	6.31 ± 0.55	5.96 ± 0.70	6.06 ± 0.49	1.074	0.379
CH5	8.82 ± 7.33	7.49 ± 3.14	5.49 ± 4.68	5.56 ± 4.56	6.04 ± 4.10	7.41 ± 3.99	1.510	0.912	CH24	6.15 ± 0.17	6.36 ± 0.94	6.11 ± 1.10	6.10 ± 0.55	7.30 ± 1.63	5.20 ± 0.89	8.882	0.041
CH6	7.48 ± 5.77	5.05 ± 4.75	6.09 ± 2.83	6.39 ± 4.82	5.19 ± 5.56	5.69 ± 5.15	0.664	0.651	CH25	6.11 ± 0.98	13.94 ± 18.37	6.09 ± 0.76	6.15 ± 0.72	7.65 ± 1.64	6.08 ± 0.44	3.444	0.006
CH7	6.07 ± 1.15	6.28 ± 0.59	6.03 ± 0.76	6.55 ± 1.25	6.27 ± 1.32	6.20 ± 1.01	0.629	0.678	CH26	6.35 ± 0.73	5.84 ± 1.45	6.37 ± 1.43	6.54 ± 1.03	5.80 ± 1.42	6.21 ± 1.27	1.183	0.322
CH8	6.61 ± 2.07	6.41 ± 0.68	5.82 ± 0.71	6.28 ± 0.83	6.03 ± 0.68	6.19 ± 1.09	1.228	0.301	CH27	5.57 ± 0.94	8.69 ± 1.38	8.49 ± 2.01	7.79 ± 1.97	6.18 ± 1.95	6.07 ± 2.66	10.20	0.945
CH9	6.51 ± 6.25	8.14 ± 5.27	4.84 ± 4.07	6.75 ± 6.21	8.81 ± 9.38	7.27 ± 7.68	0.858	0.512	CH28	4.73 ± 2.81	7.08 ± 1.52	6.05 ± 0.98	6.32 ± 0.98	5.94 ± 1.03	5.95 ± 0.55	5.142	0.013
CH10	6.09 ± 0.78	6.42 ± 0.95	6.59 ± 1.44	6.51 ± 1.94	5.87 ± 1.21	6.55 ± 3.27	0.518	0.762	CH29	7.02 ± 2.28	6.47 ± 1.67	6.39 ± 1.19	6.57 ± 1.10	6.07 ± 1.25	6.49 ± 1.29	0.821	0.537
CH11	6.18 ± 0.54	6.49 ± 1.27	6.43 ± 1.11	6.27 ± 1.07	6.98 ± 1.62	6.95 ± 1.48	1.531	0.186	CH30	6.49 ± 0.75	6.49 ± 0.75	6.77 ± 1.80	6.89 ± 1.82	6.84 ± 2.60	6.40 ± 1.50	0.316	0.902
CH12	6.11 ± 0.55	6.27 ± 0.68	5.99 ± 0.71	6.38 ± 0.69	6.22 ± 0.84	6.26 ± 0.59	0.800	0.552	CH31	6.13 ± 0.55	6.37 ± 0.80	6.57 ± 1.69	5.75 ± 2.60	6.67 ± 2.17	6.98 ± 4.17	0.687	0.634
CH13	6.42 ± 1.46	7.60 ± 3.06	6.98 ± 3.13	7.95 ± 6.37	5.96 ± 3.46	7.22 ± 3.30	0.776	0.569	CH32	6.13 ± 5.53	6.37 ± 1.07	6.26 ± 2.19	6.66 ± 2.98	6.99 ± 3.69	6.14 ± 0.74	0.468	0.799
CH14	6.41 ± 0.55	6.57 ± 0.93	6.13 ± 0.93	6.31 ± 0.88	6.23 ± 0.93	6.45 ± 0.46	0.713	0.615	CH33	6.36 ± 1.04	6.76 ± 1.12	6.55 ± 1.58	6.78 ± 0.97	6.13 ± 1.47	6.25 ± 1.47	0.868	0.505
**CH15**	6.23 ± 0.93	11.11 ± 5.03	6.15 ± 0.70	6.37 ± 0.70	5.92 ± 0.69	6.16 ± 0.92	1.719	0.003	CH34	6.17 ± 0.69	6.26 ± 0.52	6.14 ± 0.61	6.48 ± 0.66	5.93 ± 1.34	6.27 ± 0.97	0.912	0.476
CH16	6.52 ± 1.39	6.93 ± 1.72	7.38 ± 4.28	5.89 ± 1.17	6.46 ± 1.72	6.84 ± 2.91	0.848	0.518	CH35	6.10 ± 0.71	6.16 ± 0.78	6.03 ± 1.13	6.79 ± 1.26	6.05 ± 1.02	6.50 ± 2.85	0.854	0.514
CH17	6.05 ± 1.07	6.59 ± 0.69	6.25 ± 0.86	6.40 ± 0.58	6.02 ± 0.64	6.94 ± 3.07	1.166	0.330	CH36	6.66 ± 1.16	6.34 ± 0.83	6.16 ± 1.13	6.41 ± 0.80	6.54 ± 1.42	5.35 ± 4.70	0.933	0.462
**CH18**	6.29 ± 0.78	8.62 ± 4.14	6.50 ± 2.25	6.75 ± 1.40	5.89 ± 0.85	6.22 ± 1.16	4.270	0.010	CH37	6.35 ± 0.59	5.98 ± 0.79	6.18 ± 0.53	6.29 ± 0.79	5.96 ± 1.16	5.30 ± 4.18	0.839	0.524
CH19	6.98 ± 2.73	7.03 ± 1.80	6.47 ± 0.71	6.79 ± 1.73	6.07 ± 1.28	6.43 ± 2.49	1.002	0.420									

^*^The *p*-values in the figure are the results after FDR correction.

### 3.2 Changes in in prefrontal cortex FC at different time points before and after iTBS

**Figure 4:**
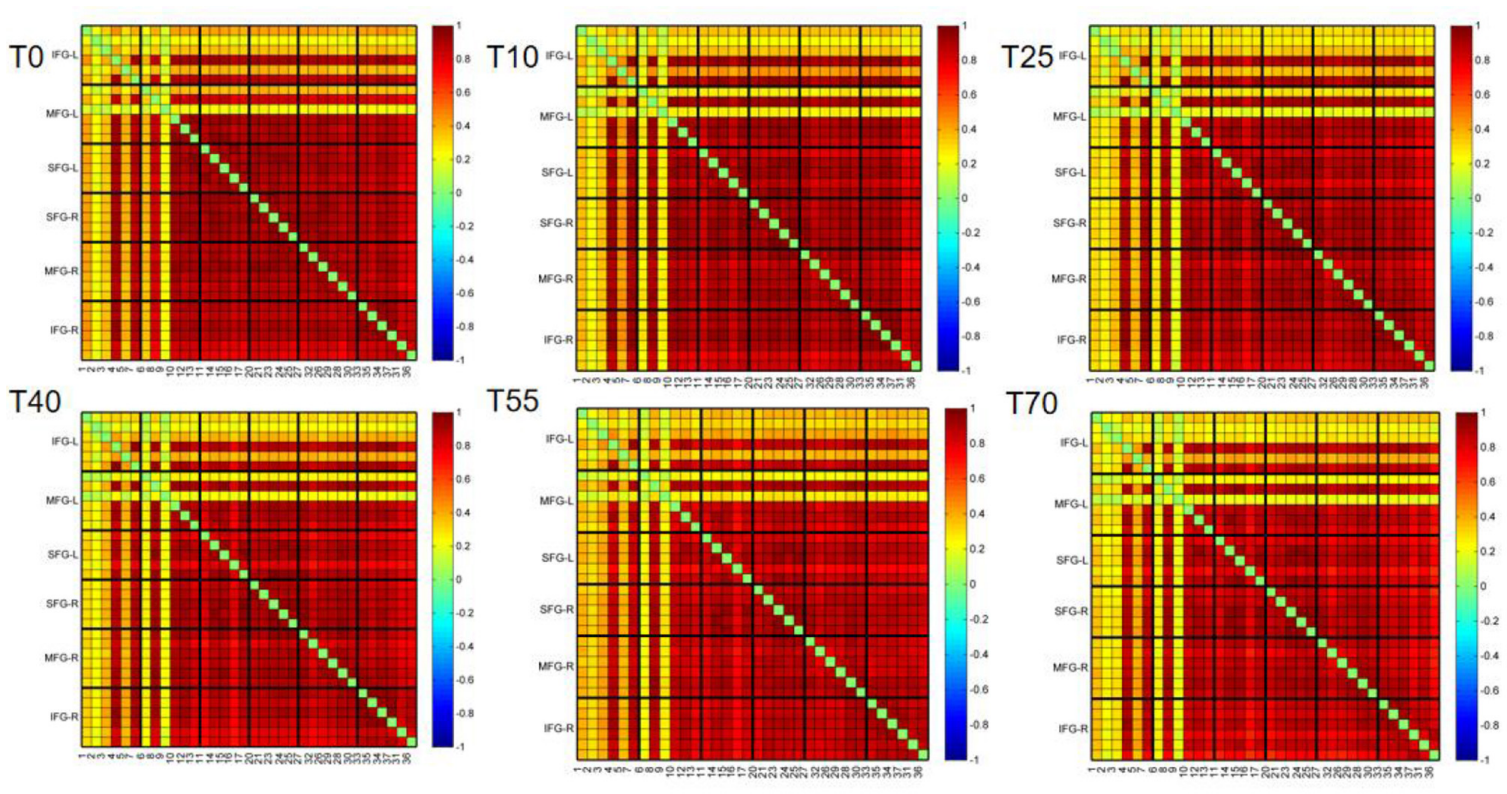
Group-average functional connectivity matrices of the prefrontal cortex at rest before (T0) and at 10, 25, 40, 55, and 70 min after iTBS stimulation. The panels from left to right and top to bottom are IFG-L, MFG-L, SFG-L, SFG-R, MFG-R, IFG-R, and the corresponding channels, respectively. Red indicates strong functional connectivity between channels, while blue indicates weak functional connectivity between channels.

To observe differences in FC in the prefrontal cortex during resting state before and after TMS intervention, we used Pearson correlation coefficients to calculate correlations between channels. The resulting FC matrix was shown in Figure 4. The FC matrix directly reflected the connection strength between channels in the monitored brain region. The 37 × 37 matrix represents connection strengths among 37 channels. Because the FC matrix is symmetric, we analyzed only the lower triangular part, excluding the diagonal elements (self-connections) and the redundant upper triangular portion. Figure 4 showed that at different time points before and after iTBS intervention, FC intensity was strong in the bilateral superior frontal gyrus (SFG), middle frontal gyrus (MFG), and right inferior frontal gyrus (IFG). In contrast, FC intensity between the left IFG (IFG-L) and other brain regions was relatively weak.

**Figure 5:**
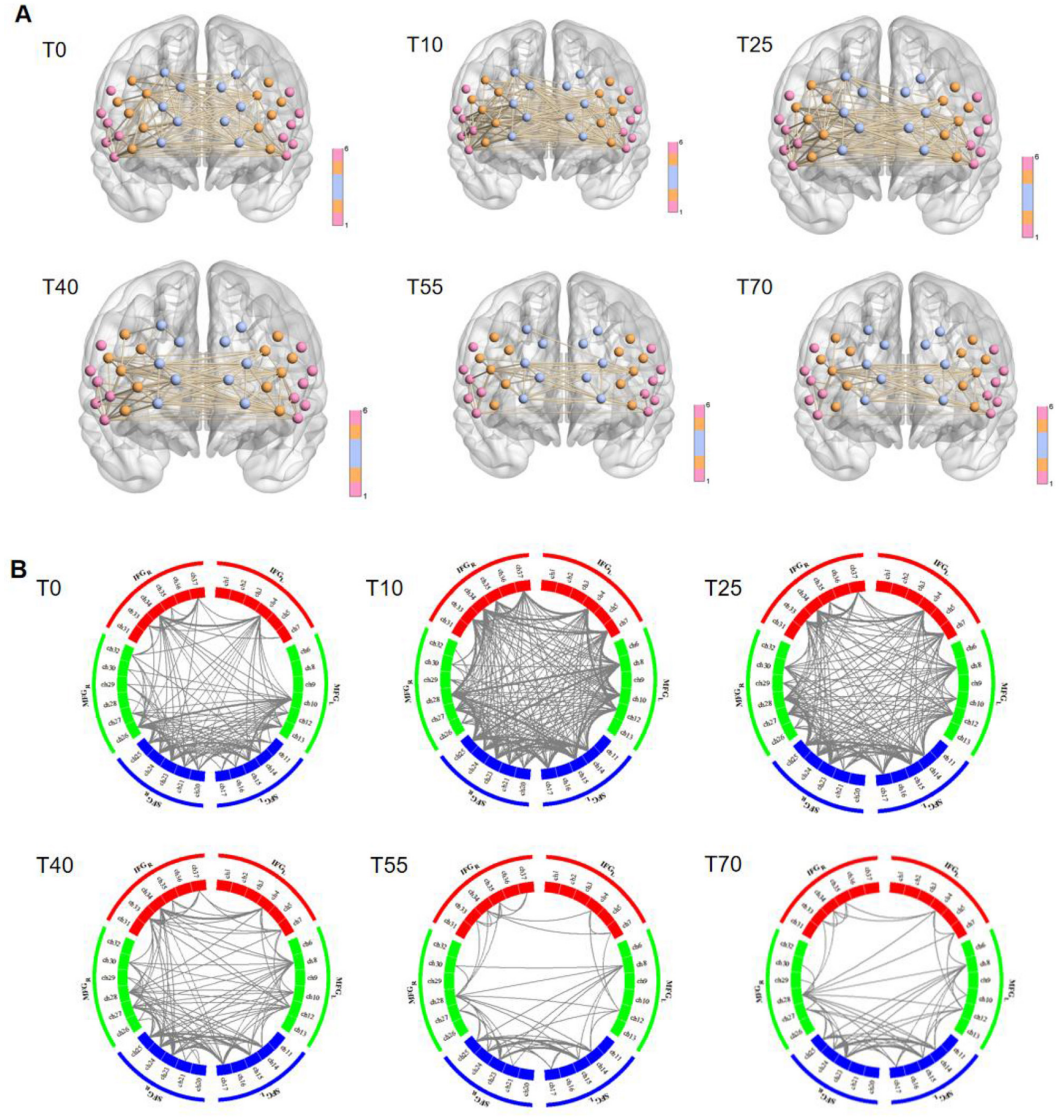
Visualization of group-average functional connectivity strength in the prefrontal cortex at rest before (T0) and 10, 25, 40, 55, and 70 min after iTBS stimulation. **(A)** 3D diagram. Nodes represent channels, with colors used to distinguish brain regions; lines indicate functional connectivity between channels. **(B)** Circle diagram. The circular region represents the prefrontal cortex, and colors (such as red, green, and blue) are used to distinguish brain regions within functional groups. Gray lines between brain regions indicate connection relationships between them.

**Figure 6:**
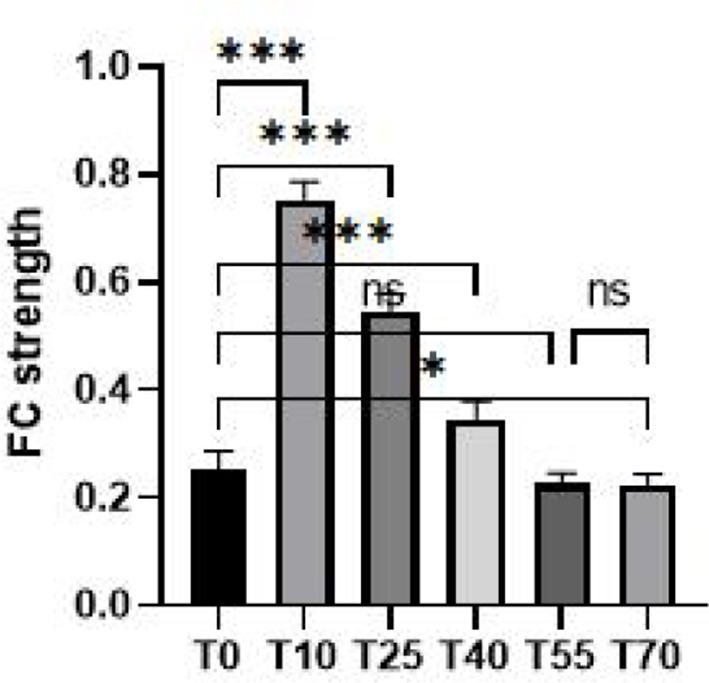
Quantification maps of group-average FC strength in the prefrontal cortex at rest before (T0) and 10, 25, 40, 55, and 70 min after iTBS stimulation. *p < 0.05, ***p < 0.001.

To further investigate differences in FC in the prefrontal cortex at different time points before and after iTBS stimulation, we first selected detection channels with statistically significant differences. Then, we plotted 3D images of brain network FC between these channels and their corresponding brain regions (Figure 5A), as well as circle plots (Figure 5B). Based on the observation of channel coverage locations, FC was significantly enhanced during the T10 period compared to T0, both between key channels and their corresponding brain regions. Statistical results for FC intensity (Figure 6) showed that FC intensity was significantly elevated in the early stage (T10) following a single iTBS intervention compared to baseline (T0). However, this effect clearly decayed over time: there was no difference between the T55 and T0 periods, and FC intensity returned to baseline levels by T55. Although FC intensity in the T70 period was lower than in the T55 period, there was no statistically significant difference between the two. These results confirm that the neuromodulatory effect of a single iTBS intervention lasts up to 40 min, providing a key time window for optimizing clinical treatment intervals.

## 4 Discussion

Repetitive transcranial magnetic stimulation (rTMS) technology, including intermittent theta burst stimulation (iTBS), has been shown to significantly improve cognitive function and activities of daily living in stroke patients (Xu et al., 2024; Yu et al., 2024; Han et al., 2023). Among these, stimulation targeting the left dorsolateral prefrontal cortex (DLPFC) has emerged as an effective strategy for treating cognitive impairments. However, in general, the effects of long-term interventions are cumulative immediate effects, and a single effect tends to reflect the direction of the effects of long-term interventions (Walther et al., 2020; Chiang et al., 2007). The present study thus looks at the single effect of iTBS in order to inform the application of iTBS. The findings of this study indicate that a single iTBS stimulation of the left DLPFC in healthy subjects resulted in an increase in blood volume and enhanced functional connectivity in the prefrontal cortex of the brain. This was evidenced by an increase in HbO_2_ activation and a greater strength of functional connectivity after the stimulation compared with the pre-stimulation period of T0, which persisted until 40 min after the stimulation.

iTBS is a non-invasive brain stimulation technique designed to enhance neuroplasticity in specific areas of the brain through short bursts of high-frequency stimulation. Stimulation targeting the left DLPFC may increase the excitability of this region, thereby affecting related neural network functions. The results of our study indicate that blood volume in the prefrontal cortex of the brain increased following iTBS stimulation. This finding suggests that the region may exhibit heightened metabolic activity, which is commonly associated with neuronal activity. The increased HbO_2_ levels observed in our study imply that more oxygen is delivered to active neural regions, which may be associated with the increased energy demands of neurons. An increase in blood flow facilitates the delivery of oxygen and nutrients to support neuronal activity and synaptic transmission. Furthermore, the enhancement of FC following iTBS stimulation suggests an increase in information transfer and synergy between brain regions. This may be attributed to enhanced interactions between the DLPFC and other associated regions (e.g., bilateral SFGs, MFGs, and right IFGs). The DLPFC, as a pivotal hub for executive function and cognitive control, may facilitate more intricate cognitive processes by modulating connectivity between these regions. In addition, it was found that the correlation between IFG-L and other brain regions was not significant, which may reflect the independence of this region in specific functions or cognitive tasks, as the IFG is commonly associated with language processing and social cognition (Obayashi, 2022).

There are relatively few experimental studies of the behavior of rTMS in healthy individuals. (Vanderhasselt et al. 2006) applied a single 10 Hz high-frequency repetitive rTMS to stimulate the DLPFC of healthy young females. After high-frequency rTMS, healthy young female subjects demonstrated enhanced performance on the Stroop task compared to those who received sham stimulation. (Hwang et al. 2010) conducted a similar study with healthy young males and found that healthy young males made fewer errors on the Conners Continuous Task Test after high-frequency rTMS. Nevertheless, the findings of (Rounis et al. 2006) indicated that a single session of high-frequency rTMS to the left DLPFC did not result in an improvement in attentional orientation in healthy young individuals (both male and female). According to the previous literature (Guse et al., 2010), after rTMS intervention, healthy subjects are less likely to produce effects than patients. The disparate outcomes of rTMS on cognitive control may be attributable to the discrepancies in rTMS parameters employed across the aforementioned studies, including the type of stimulus, its location, frequency, intensity, and duration. It is widely accepted that low-frequency rTMS ( ≤ 1 Hz) has the capacity to inhibit the firing activity of the stimulated local neurons and reduce the excitability of the nerve cells (Lefaucheur et al., 2020; Buetefisch et al., 2023). Conversely, high-frequency rTMS (5–25 Hz) has the effect of depolarising and increasing the excitability of the stimulated local nerve cells, which results in a notable enhancement of neurological function and cognitive improvement (Lefaucheur et al., 2020; Pell et al., 2011).

This study innovatively combines fNIRS with iTBS. However, superficial artifacts in fNIRS may confound iTBS-induced hemodynamic changes in several ways (Nambu et al., 2017; Izzetoglu and Holtzer, 2020). First, the temporal distribution of hemodynamic changes in superficial tissues resembles typical cerebral hemodynamic models, and fluctuations caused by bodily movements may overlap with iTBS-induced cerebral changes. Second, fNIRS signals have complex origins, and local hemodynamic changes in superficial extracerebral tissues can act as significant noise, potentially leading to misinterpretation as iTBS-induced brain activation. Additionally, physiological changes such as heart rate and blood pressure induced by iTBS may overlap with superficial tissue hemodynamics, complicating the signal and obscuring or distorting the true iTBS effects. To address this issue, we implemented superficial signal regression and short separation correction techniques on fNIRS data to address contamination from extracranial hemodynamics.

This study has several limitations. First, the relatively small sample size may affect the generalizability and statistical power of the results. Second, resting-state functional connectivity based on fNIRS is limited to the prefrontal cortex and related regions, and cannot provide data from subcortical areas. Third, due to the limited number of signal channels available in current fNIRS devices, we only analyzed a specific brain network with 37 channels. Additionally, the study examined only the immediate effects after a single intervention, and functional connectivity was based on time-domain signals from regions of interest. This approach may have overlooked secondary connections between other brain regions. Future studies should increase the number of channels and expand the sample size to more comprehensively assess the impact of iTBS on brain function.

## Data Availability

The raw data supporting the conclusions of this article will be made available by the authors, without undue reservation.

## References

[B1] Beam W. Borckardt J. J. Reeves S. T. George M. S. (2009). An efficient and accurate new method for locating the F3 position for prefrontal TMS applications. Brain Stimul. 2, 50–54. 10.1016/j.brs.2008.09.006 20539835 PMC2882797

[B2] Blumberger D. M. Vila-Rodriguez F. Thorpe K. E. Feffer K. Noda Y. Giacobbe P.. (2018). Effectiveness of theta burst versus high-frequency repetitive transcranial magnetic stimulation in patients with depression (THREE-D): a randomised non-inferiority trial. Lancet 391, 1683–1692. 10.1016/S0140-6736(18)30295-2 29726344

[B3] Buetefisch C. M. Wei L. Gu X. Epstein C. M. Yu S. P. (2023). Neuroprotection of low-frequency repetitive transcranial magnetic stimulation after ischemic stroke in rats. Ann. Neurol. 93, 336–347. 10.1002/ana.26509 36097798 PMC10042643

[B4] Chen D. Y. Di X. Yu X. Biswal B. B. (2023). The significance and limited influence of cerebrovascular reactivity on age and sex effects in task- and resting-state brain activity. bioRxiv. 10.1101/2023.08.18.553848 38212284 PMC10832986

[B5] Chen H. Miao G. Wang S. Zheng J. Zhang X. Lin J.. (2023). Disturbed functional connectivity and topological properties of the frontal lobe in minimally conscious state based on resting-state fNIRS. Front. Neurosci. 17:1118395. 10.3389/fnins.2023.1118395 36845431 PMC9950516

[B6] Chiang T. C. Vaithianathan T. Leung T. Lavidor M. Walsh V. Delpy D. T.. (2007). Elevated haemoglobin levels in the motor cortex following 1 Hz transcranial magnetic stimulation: a preliminary study. Exp. Brain Res. 181, 555–560. 10.1007/s00221-007-0952-x 17530233

[B7] Cui X. Bray S. Reiss A. L. (2010). Functional near infrared spectroscopy (NIRS) signal improvement based on negative correlation between oxygenated and deoxygenated hemoglobin dynamics. Neuroimage 49, 3039–3046. 10.1016/j.neuroimage.2009.11.050 19945536 PMC2818571

[B8] Dong L. Chen W. C. Su H. Wang M. L. Du C. Jiang X. R.. (2023). Intermittent theta burst stimulation to the left dorsolateral prefrontal cortex improves cognitive function in polydrug use disorder patients: a randomized controlled trial. Front Psychiatry 14:1156149. 10.3389/fpsyt.2023.1156149 37304431 PMC10248467

[B9] Guse B. Falkai P. Wobrock T. (2010). Cognitive effects of high-frequency repetitive transcranial magnetic stimulation: a systematic review. J. Neural Transm. 117, 105–122. 10.1007/s00702-009-0333-7 19859782 PMC3085788

[B10] Han M. He J. Chen N. Gao Y. Wang Z. Wang K.. (2023). Intermittent theta burst stimulation vs. *h*igh-frequency repetitive transcranial magnetic stimulation for post-stroke cognitive impairment: protocol of a pilot randomized controlled double-blind trial. Front Neurosci. 17:1121043. 10.3389/fnins.2023.1121043 37065916 PMC10098089

[B11] Hu L. He J. Han M. Wang Z. Gao Y. Zhang B.. (2025). Single high-frequency repetitive transcranial magnetic stimulation and intermittent theta pulse stimulation promote working memory behavior in participants: an event-related potential study. Brain Res. Bull. 220:111147. 10.1016/j.brainresbull.2024.111147 39608615

[B12] Hwang J. H. Kim S. H. Park C. S. Bang S. A. Kim S. E. (2010). Acute high-frequency rTMS of the left dorsolateral prefrontal cortex and attentional control in healthy young men. Brain Res. 1329, 152–158. 10.1016/j.brainres.2010.03.013 20226772

[B13] Izzetoglu M. Holtzer R. (2020). Effects of processing methods on fNIRS signals assessed during active walking tasks in older adults. IEEE Trans. Neural Syst. Rehabil. Eng. 28, 699–709. 10.1109/TNSRE.2020.2970407 32070987 PMC7768789

[B14] Jiang T. Wang M. Hao X. Xu J. Zhang Q. Wei X.. (2023). Intermittent theta burst stimulation for poststroke non-spatial attention deficit: a protocol of prospective, double-blinded, single-centre, randomised controlled trial in China. BMJ Open. 13:e075131. 10.1136/bmjopen-2023-075131 37816555 PMC10565327

[B15] Knecht S. Deppe M. Dräger B. Bobe L. Lohmann H. Ringelstein E.. (2000). Language lateralization in healthy right-handers. Brain. 123(Pt 1), 74–81. 10.1093/brain/123.1.74 10611122

[B16] Kong Y. Zhou J. Zhao M. Zhang Y. Tan T. Xu Z.. (2023). Non-inferiority of intermittent theta burst stimulation over the left V1 vs. classical target for depression: a randomized, double-blind trial. J. Affect. Disord. 343, 59–70. 10.1016/j.jad.2023.09.024 37751801

[B17] Lefaucheur J. P. Aleman A. Baeken C. Benninger D. H. Brunelin J. Di Lazzaro V. (2020). Evidence-based guidelines on the therapeutic use of repetitive transcranial magnetic stimulation (rTMS): an update (2014-2018). Clin. Neurophysiol. 131, 474–528. 10.1016/j.clinph.2019.11.002 31901449

[B18] Li X. Qi G. Yu C. Lian G. Zheng H. Wu S.. (2021). Cortical plasticity is correlated with cognitive improvement in Alzheimer's disease patients after rTMS treatment. Brain Stimul. 14, 503–510. 10.1016/j.brs.2021.01.012 33581283

[B19] Liu Y. Luo J. Fang J. Yin M. Cao J. Zhang S.. (2023). Screening diagnosis of executive dysfunction after ischemic stroke and the effects of transcranial magnetic stimulation: a prospective functional near-infrared spectroscopy study. CNS Neurosci. Ther. 29, 1561–1570. 10.1111/cns.14118 36786133 PMC10173709

[B20] Luo Y. Du J. Yu H. Fang F. Shi P. (2024). Resting-state fNIRS reveals changes in prefrontal cortex functional connectivity during TENS in patients with chronic pain. Sci. Rep. 14:29187. 10.1038/s41598-024-79820-2 39587185 PMC11589569

[B21] Nambu I. Ozawa T. Sato T. Aihara T. Fujiwara Y. Otaka Y.. (2017). Transient increase in systemic interferences in the superficial layer and its influence on event-related motor tasks: a functional near-infrared spectroscopy study. J. Biomed. Opt. 22:35008. 10.1117/1.JBO.22.3.035008 28294282

[B22] Obayashi S. (2022). Cognitive and linguistic dysfunction after thalamic stroke and recovery process: possible mechanism. AIMS Neurosci. 9, 1–11. 10.3934/Neuroscience.2022001 35434274 PMC8941189

[B23] Pell G. S. Roth Y. Zangen A. (2011). Modulation of cortical excitability induced by repetitive transcranial magnetic stimulation: influence of timing and geometrical parameters and underlying mechanisms. Prog. Neurobiol. 93, 59–98. 10.1016/j.pneurobio.2010.10.003 21056619

[B24] Pinti P. Tachtsidis I. Hamilton A. Hirsch J. Aichelburg C. Gilbert S.. (2020). The present and future use of functional near-infrared spectroscopy (fNIRS) for cognitive neuroscience. Ann NY Acad Sci. 1464, 5–29. 10.1111/nyas.13948 30085354 PMC6367070

[B25] Rounis E. Stephan K. E. Lee L. Siebner H. R. Pesenti A. Friston K. J.. (2006). Acute changes in frontoparietal activity after repetitive transcranial magnetic stimulation over the dorsolateral prefrontal cortex in a cued reaction time task. J. Neurosci. 26, 9629–9638. 10.1523/JNEUROSCI.2657-06.2006 16988033 PMC6674444

[B26] Sowell E. R. Peterson B. S. Thompson P. M. Welcome S. E. Henkenius A. L. Toga A. W.. (2003). Mapping cortical change across the human life span. Nat. Neurosci. 6, 309–315. 10.1038/nn1008 12548289

[B27] Struckmann W. Bodén R. Gingnell M. Fällmar D. Persson J. (2022). Modulation of dorsolateral prefrontal cortex functional connectivity after intermittent theta-burst stimulation in depression: combining findings from fNIRS and fMRI. Neuroimage Clin. 34:103028. 10.1016/j.nicl.2022.103028 35537216 PMC9118162

[B28] Su H. Chen T. Jiang H. Zhong N. Du J. Xiao K.. (2020). Intermittent theta burst transcranial magnetic stimulation for methamphetamine addiction: a randomized clinical trial. Eur. Neuropsychopharmacol. 31, 158–161. 10.1016/j.euroneuro.2019.12.114 31902567

[B29] Tamnes C. K. Ostby Y. Fjell A. M. Westlye L. T. Due-Tønnessen P. Walhovd K. B.. (2010). Brain maturation in adolescence and young adulthood: regional age-related changes in cortical thickness and white matter volume and microstructure. Cereb. Cortex. 20, 534–48. 10.1093/cercor/bhp118 19520764

[B30] Vanderhasselt M. A. De Raedt R. Baeken C. Leyman L. D'haenen H. (2006). The influence of rTMS over the left dorsolateral prefrontal cortex on stroop task performance. Exp Brain Res. 169, 279–282. 10.1007/s00221-005-0344-z 16418843

[B31] Walther S. Kunz M. Müller M. Zürcher C. Vladimirova I. Bachofner H.. (2020). Single session transcranial magnetic stimulation ameliorates hand gesture deficits in schizophrenia. Schizophr. Bull. 46, 286–293. 10.1093/schbul/sbz078 31634401 PMC7442336

[B32] Wang S. Lu S. (2022). Brain functional connectivity in the resting state and the exercise state in elite tai chi chuan athletes: an fNIRS study. Front. Hum. Neurosci. 16:913108. 10.3389/fnhum.2022.913108 35782040 PMC9243259

[B33] Webler R. D. Fox J. McTeague L. M. Burton P. C. Dowdle L. Short E. B.. (2022). DLPFC stimulation alters working memory related activations and performance: an interleaved TMS-fMRI study. Brain Stimul. 15, 823–832. 10.1016/j.brs.2022.05.014 35644517

[B34] Wu X. Yan Y. Hu P. Wang L. Wu Y. Wu P.. (2024). Effects of a periodic intermittent theta burst stimulation in Alzheimer's disease. Gen Psychiatr. 37:e101106. 10.1136/gpsych-2023-101106 38274292 PMC10806514

[B35] Xia M. Yang S. Simpkins J. W. Liu H. (2007). Noninvasive monitoring of estrogen effects against ischemic stroke in rats by near-infrared spectroscopy. Appl. Opt. 46, 8315–8321. 10.1364/AO.46.008315 18059674

[B36] Xu B. Lin C. Wang Y. Wang H. Liu Y. Wang X.. (2024). Using dual-target rTMS, single-target rTMS, or Sham rTMS on post-stroke cognitive impairment. J. Integr. Neurosci. 23:161. 10.31083/j.jin2308161 39207080

[B37] Yu H. Shu X. Zhou Y. Zhou S. Wang X. (2024). Intermittent theta burst stimulation combined with cognitive training improves cognitive dysfunction and physical dysfunction in patients with post-stroke cognitive impairment. Behav. Brain Res. 461:114809. 10.1016/j.bbr.2023.114809 38081516

